# Physical space of thirty pediatric intensive care units in the United States of America: a national survey

**DOI:** 10.3389/fped.2024.1473805

**Published:** 2024-09-18

**Authors:** Oliver Karam, Aziez Ahmed, Matthew Bizzarro, Clifford Bogue, John S. Giuliano, PICU Space Collaborative, Awni Al-Subu

**Affiliations:** ^1^ Department of Pediatrics, Pediatric Critical Care Medicine, Yale School of Medicine, New Haven, CT, United States; ^2^ Department of Pediatrics, Neonatal-Perinatal Medicine, Yale School of Medicine, New Haven, CT, United States

**Keywords:** hospitals, patient care, intensive care units, pediatric, surveys and questionnaires, personal satisfaction

## Abstract

**Introduction:**

The design of Pediatric Intensive Care Unit (PICU) rooms significantly impacts patient care and satisfaction. The aims were first, to describe the current physical space across PICUs in the USA, and second, to identify what proportion of PICUs are compliant with current guidelines.

**Methods:**

A descriptive cross-sectional survey was conducted, targeting division chiefs and medical directors of PICUs nationwide. The survey collected data on unit type, construction and renovation dates, room sizes, and available amenities. According to the Guidelines for Design and Construction of Hospitals, PICU rooms are recommended to be single rooms, at least 200 sq ft, have a window and a private bathroom. Data were anonymized and reported as median and interquartile ranges or frequencies and percentages.

**Results:**

Thirty units responded. Among the respondents, 26 had general PICUs, 9 had cardiac ICUs, and 3 had intermediate care units, with some units containing multiple types of ICUs. The median annual admissions were 1,125, with a median occupancy rate of 78%. Twenty-three percent of units had at least one double room, and 3% had triple or quadruple rooms. The median room size was 265 sq ft (IQR 230; 304), the smallest room size was 220 sq ft (IQR 179; 275), and the largest single room size was 312 sq ft (IQR 273; 330). Thirty-seven percent of units had bathrooms in every room, while 80% had windows in every room. Additionally, 46% of units had dialysis capabilities in every room, and 7% had negative pressure capabilities in every room. The median building year was 2008 (IQR 2001;2014), with 36% of units having undergone at least one renovation. Larger rooms were associated with more recent build dates (*p* = 0.01). Only 30% of the PICUs met the guidelines for physical space. These compliant units were built at a median of 4 years ago (IQR 1; 8).

**Conclusion:**

This study highlights the variability in PICU room design and amenities across healthcare facilities. Many units still fall short of meeting the guidelines for room size, windows, and private bathrooms. Future research should investigate the relationship between room characteristics and patient outcomes to inform better design practices, with a goal of improving patient experiences and clinical outcomes.

## Introduction

The design and features of Pediatric Intensive Care Unit (PICU) rooms are crucial for patient care, impacting clinical outcomes and patient and family satisfaction (1). Recent studies have highlighted the importance of room size, amenities, and environmental factors in contributing to optimal patient care and infection control (1, 2). The evolving standards of care and technological advancements necessitate periodic assessments of PICU infrastructure to ensure the needs of critically ill children and current standards are met (3).

Effective PICU room sizes and layouts enhance workflow and improve communication, which are critical in high-stress environments (4). Optimized spatial design and strategic placement of equipment and supply rooms minimize staff fatigue and streamline tasks (5, 6). These design improvements (7) can create sustainable work conditions in PICUs that ultimately may help to mitigate the high burnout rates commonly observed among pediatric intensivists (8). Evidence from studies on primary care providers has shown that higher satisfaction with the physical work environment is associated with reduced burnout rates, suggesting that similar benefits could be realized in pediatric critical care settings (9).

Sufficient physical space is crucial for ensuring flexibility and adaptability in PICU room design. The increasing number of monitoring devices (near-infrared spectroscopy, video electroencephalogram) and life-support technologies, such as continuous renal replacement therapy, high-frequency oscillation, and extracorporeal membrane oxygenation (ECMO), demand additional space and infrastructure. Sufficient space and flexible designs allow for quick adjustments to accommodate these devices, ensuring rooms can integrate new equipment seamlessly without extensive renovations.

However, there is a paucity of comprehensive data comparing these characteristics across different types of PICU rooms, including general PICUs, Cardiac Intensive Care Units (CICUs), and Intermediate Care Units, especially in relation to the impact of recent construction and renovations.

This study aims to fill this gap by providing an in-depth description of the physical attributes and amenities of PICU rooms across a wide range of healthcare facilities. By examining factors such as room size, access to private bathrooms and other essential amenities, and the year of construction or latest renovation, this research seeks to identify trends and disparities that could inform future design and renovation projects.

## Methods

### Study design

This study employed a descriptive, cross-sectional survey design to assess the characteristics and amenities of Pediatric Intensive Care Unit (PICU) rooms across various healthcare facilities.

### Ethics

This project met specified criteria for a quality improvement initiative and was therefore deemed exempt from review by our institutional review board.

### Survey development

An online survey was developed to capture comprehensive information about PICU rooms, including the type of unit, construction and renovation dates, room sizes, and available amenities. The survey was piloted in three units, to ensure consistency.

### Survey distribution

We targeted division chiefs and medical directors of PICUs nationwide. We disseminated the survey through email invitations and reminders to division chiefs and medical directors of PICUs around the country. In addition to the survey being published on the Pediatric Critical Care Chiefs Network (pc3n.org), direct emails were sent to the authors’ personal contacts, acknowledging there might be an overlap. The survey was accessible for completion over a four-week period, with weekly reminders sent to encourage participation.

### Variable definitions

For the purpose of this study, we defined several key variables to systematically describe the PICU rooms, such as the size of the smallest room, the size of the largest room, the average room size for the unit, the percentage of single rooms, the presence of windows, and bathrooms. The responders were encouraged to use construction blueprints or floor plans to report room sizes. The average room size was calculated by adding the sizes of all rooms and dividing by the number of rooms.

The units were described in terms of total annual admissions, average daily census and occupancy rate. The “years since built or renovation” was calculated as the number of years from the most recent construction or renovation event to present, distinguishing between units that were newly built and those that had undergone renovations.

Most states in the USA use the Guidelines for Design and Construction of Hospitals, published every four years by the Facility Guidelines Company (3). These guidelines, last published in 2022, recommend that each pediatric critical care room be single occupancy, have at least 200 sq ft (18.6 m^2^), have a window with exterior light, a private bathroom, and a dedicated space for parents to stay. Additionally, rooms where ECMO procedures are performed should be at least 300 sq ft (27.9 m^2^). In our study, compliance with these guidelines was calculated as the proportion of units where all rooms were single, had more than 200 sq ft, and had a window and a bathroom.

### Statistical analysis

Upon collection, survey data were anonymized and aggregated for analysis. Median and interquartile ranges (IQR) were reported for non-normally distributed continuous variables, while frequencies and percentages were used for categorical variables.

We categorized PICU units based on the quartile of average room size and analyzed the association between room size and other variables using the Kruskal-Wallis test for continuous non-parametric data. Additionally, we employed linear regression models to explore the influence of various factors on room size, including unit type, years since construction or renovation, and room amenities. Variables identified with a *p*-value of <0.1 in the univariate analysis were included in the regression models to assess their independent association with room size. Results were considered statistically significant at a *p*-value of <0.05.

A graphical representation of all variables was achieved as a heatmap. Each variable was normalized to a percentile rank to facilitate comparison across hospitals. This transformation ensured that the values were on a uniform scale from 0 to 1, with 1 representing the highest percentile rank and 0 representing the lowest. Using a data visualization tool (Matplotlib and Seaborn libraries in Python), the normalized data were visualized as a heatmap. The x-axis of the heatmap represents the hospital units, numbered from 1 to 30, while the y-axis lists the different variables. Each cell within the heatmap corresponds to the percentile rank of a specific variable for a given hospital unit. A color gradient was applied to the heatmap to visually represent the percentile ranks. Dark blue squares were used to indicate the highest percentiles, while light yellow squares indicated the lowest percentiles. A color bar was also included to provide a reference for interpreting the color gradient in terms of percentile rank.

Data analysis was performed using SPSS 28 (Armonk, NY, USA).

## Results

The survey was distributed in January and February 2024 to division chiefs or designees participating in the Pediatric Critical Care Chiefs Network (PC3N) from 2020 to 2022.

### Units

Out of a total of 156 PICUs in the PC3N, 30 units responded to this survey (response rate: 19%). As shown in Supplementary Figure 1, there was some overlap between 26 PICUs, 9 CICUs, and 3 Intermediate Care Units. The median annual admissions were 1,125 (IQR 990;1715, Supplementary Figure 2), with an average daily census of 16 (IQR 14;26) translating into a median occupancy rate of 78% (IQR 69;88, Supplementary Figure 3).

### Building and renovation

The median year of construction was 2008 (IQR 2001;2014). Thirty percent of the units had undergone at least one renovation, after a median time of 20 years (IQR 15;25). The median renovation year was 2017 (IQR 2014;2019). The median years since new construction and/or renovation was 10 years (IQR 6;16, Supplementary Figure 4).

### Beds and rooms

The median number of beds was 21 (IQR 18;28), while the median number of rooms was 20 (IQR 18;28). Seventy percent of the units had only single rooms (Supplementary Figure 5). Twenty-three percent of the units had at least one double room, 3% of the units had a triple room, and another 3% had a quadruple room. Overall, there were 45 (6%) out of 759 beds in shared rooms. Only three of these rooms were in step-down units.

### Room sizes

**Figure 1:**
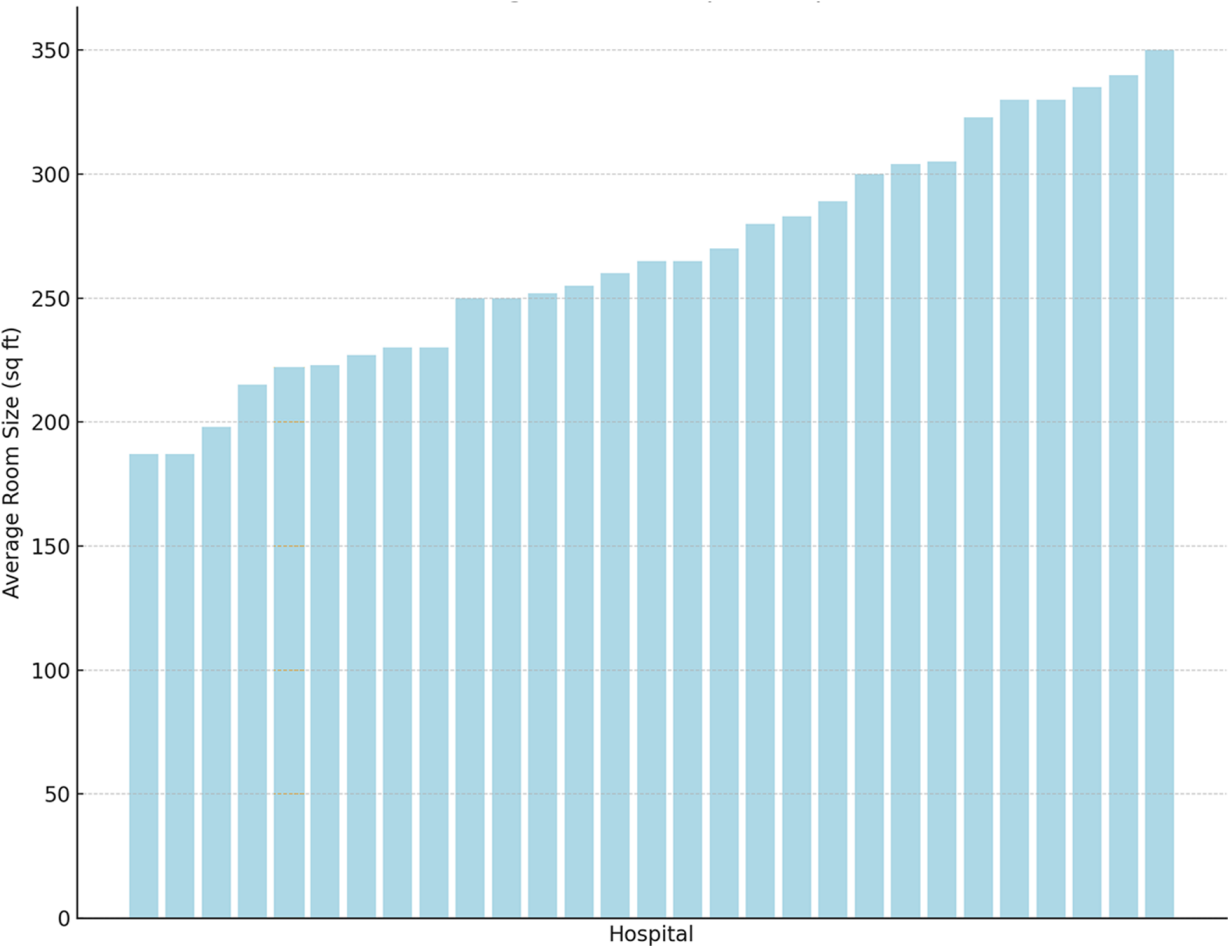
Bar chart of the average room size (in square feet, y-axis) according to the hospitals (x-axis, ranked from smallest to largest).

The median room size was 265 sq ft (IQR 230;304, Figure 1). The median size of the smallest single room was 220 sq ft (IQR 179;275, Supplementary Figure 6). Forty-three percent of the units had at least one room that was smaller than 200 sq ft. The median size of the largest single room was 312 sq ft (IQR 273;330, Supplementary Figure 7), while 424 sq ft (IQR 319;506, Supplementary Figure 8) was the largest median double room size.

In the univariate analysis, more recent build dates (*p* = 0.005) and lower occupancy rates (*p* = 0.04) were associated with larger rooms. In the regression model, only recent build date was independently associated with larger rooms (adj β 2.16, 95%CI 0.50;3.83, *p* = 0.01), indicating that for each more recent year since construction, the average size increases by 2.16 sq ft, assuming all other variables are held constant.

### Room amenities

Thirty-seven percent of the units have bathrooms in every room, while 20% of the units have no rooms with bathrooms (Supplementary Figure 9). Excluding units with bathrooms in every room, the median percentage of rooms with a bathroom was 55% (21;65).

Eighty percent of units had windows in every room (Supplementary Figure 10). Excluding units with windows in every room, the median percentage of rooms with windows was 85% (IQR 79;89).

Seven percent of the units had negative pressure capabilities in every room (Supplementary Figure 11). Excluding units with negative pressure capabilities in every room, the median percentage of rooms with negative pressure capabilities was 15% (IQR 10;23).

Forty-six percent of the units had dialysis capabilities in every room, while 3% of the units had no dialysis capabilities (Supplementary Figure 12). Excluding units with dialysis capabilities in every room, the median percentage of rooms with hemodialysis capabilities is 18% (9;26).

### Heterogeneity

**Figure 2:**
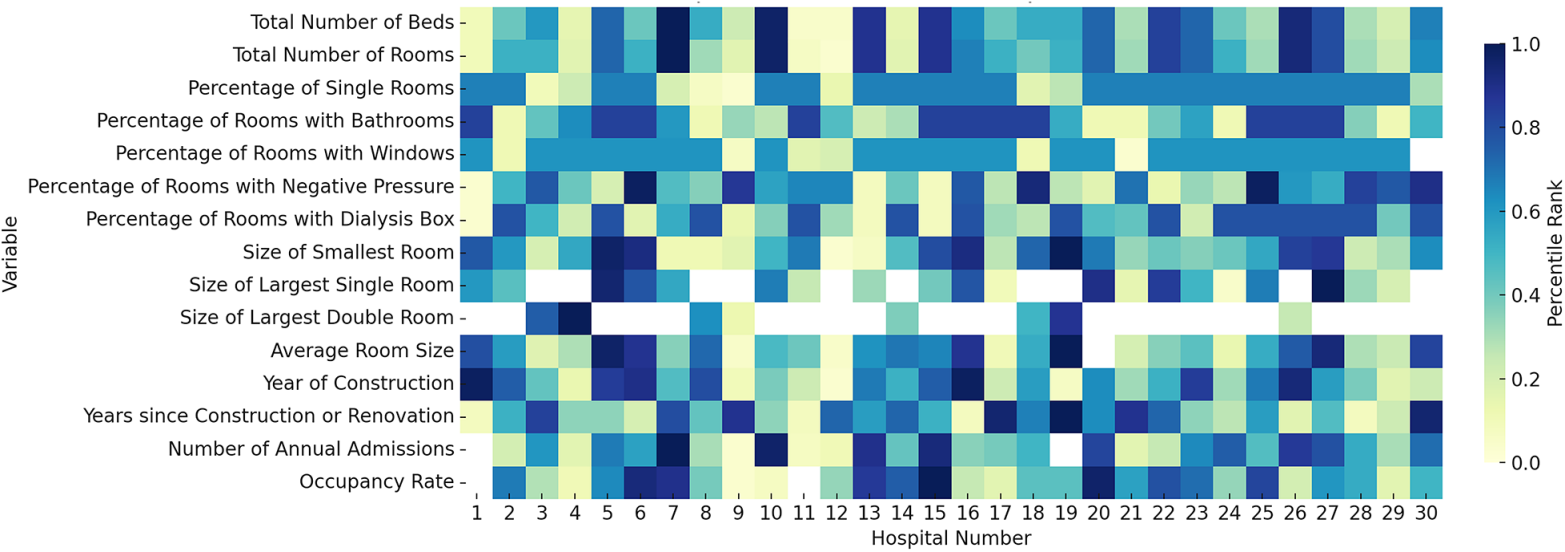
Heatmap illustrating the distribution of various variables across 30 different hospital units. The *x*-axis represents the hospital units, numbered from 1 to 30, while the y-axis lists the specific variables measured in each hospital unit. Each cell within the heatmap corresponds to the percentile rank of a particular variable for a given hospital unit. The color gradient in the heatmap indicates the percentile rank, with dark blue squares representing the highest percentiles and light yellow squares representing the lowest percentiles. For instance, in the variable “Total Number of Rooms,” hospitals numbered 7 and 10 have the highest ranks, as indicated by dark blue squares. Conversely, hospitals numbered 11 and 12 show the lowest ranks in this category, marked by light yellow squares.

Figure 2 demonstrates the heterogeneity of the units, where each hospital is ranked for each variable. This figure demonstrates that no single hospital has the largest rooms, the highest proportion of bathrooms or windows.

### Compliance with guidelines

Thirty percent of the PICUs met the guidelines for physical space (i.e., all rooms were single, had more than 200 sq ft, and had a window and a bathroom). These units were built at a median of 4 years ago (IQR 1; 8).

## Discussion

The study's findings reveal significant deficiencies in many PICUs, including adherence to current guidelines. A quarter of the units include double rooms, with some even having triple or quadruple rooms. Furthermore, although the median room size exceeds the recommended 200 square feet, half the PICUs have at least one room smaller than 200 square feet, limiting space for essential medical equipment and staff movement, as well as parental space. Additionally, one in five units lack windows in every room, which might affect patient cognitive outcomes, as some adult studies have shown an association between exposure to natural light and decreased delirium (10, 11). Finally, only one in three provide private bathrooms, which can negatively impact family comfort.

The hospital design and construction guidelines emphasize that each pediatric critical care room should be single occupancy, have at least 200 sq ft, include a window with exterior light, and provide direct access to a private bathroom (3). Additionally, rooms designated for ECMO should be over 300 sq ft. However, our study did not account for other essential factors, such as the minimal headwall width of 13 feet, clearance around the bed (5 feet at the foot and one side, 4 feet on the other side, and 1 foot at the head), space dedicated to parents that does not encroach on clearance requirements, direct visibility of each patient from the nursing station, settings that facilitate provider concentration and limit interruptions, and office space for medical and nursing leadership within the unit. Incorporating these features may be important for enhancing patient safety, improving parental satisfaction, and supporting staff well-being.

Evaluating mitigation strategies for minimizing space issues, such as moving patients to larger rooms prior to ECMO cannulation or prioritizing staff and equipment during patient resuscitation in small rooms, is challenging because these issues are rarely reported publicly. As a result, the effect of small space on critical care outcomes is unclear. Future research is necessary to understand how room characteristics influence patient outcomes, particularly in terms of post-ICU anxiety, depression, and post-traumatic stress disorder. Additionally, parental satisfaction and staff wellness, including burnout, are critical factors that need to be assessed. Understanding the relationship between room design and these outcomes can inform better practices and guidelines, ultimately improving both patient and staff experiences in PICUs.

This study has several limitations. First, the data collected is not meant to represent the ideal state but to characterize the current variation in PICU room designs. Our low response rate limits the generalizability of our findings and creates uncertainty as to whether or not the data are an accurate representation of PICUs in the USA. Additionally, there might be potential selection bias, as some units with smaller rooms may have been less inclined to share their information. Furthermore, the guidelines recommending a minimum room size of 200 sq ft were published in 1996 by the American Institute of Architects, so units designed before this may not have been aware of or adhered to these standards (12). We only inquired about rooms that had the capability of being set to negative pressure, but not those that had an antechamber, which is a feature recommended in the guidelines. Potentially other important variables such as headwall width, bed clearance, dedicated parental space, patient visibility from nursing stations, provider documentation settings, and office space for medical leadership were not collected in this survey. Finally, the study relied on self-reported data, which may be subject to reporting bias and inaccuracies. Future research should aim to address these limitations by including a more comprehensive list of design features and ensuring a more systematic and representative sample of PICUs.

In conclusion, our study highlights significant variability in PICU room features and identifies areas where some PICUs do not fully meet the current guidelines for room size, windows, and private bathrooms. These deficiencies could affect patient care quality, family comfort, and staff satisfaction. Future research should examine how these room characteristics impact clinical outcomes, including resuscitation in small rooms, patient and parental post-traumatic stress disorder, parental satisfaction, and staff wellness. Understanding the influence of specific design elements can inform better practices and enhance patient and staff experiences in PICUs.

## Data Availability

The datasets presented in this article are not readily available because the datasets generated and analyzed for this study cannot be shared publicly as they contain geographical location and proprietary census admission data that cannot be anonymized. Therefore, to protect the privacy and confidentiality of the institutions involved, the datasets cannot be made public. Requests to access the datasets should be directed to Oliver Karam, oliver.karam@yale.edu.
