# Supplementary material for: Physical space of thirty pediatric intensive care units in the United States of America: a national survey
Source: Front Pediatr. 2024 Sep 18;12:1473805. doi: 10.3389/fped.2024.1473805 (PMC11445063; doi:10.3389/fped.2024.1473805)
Supplement: Supplementary file 1 [file Datasheet1.pdf]

Supplementary Figures

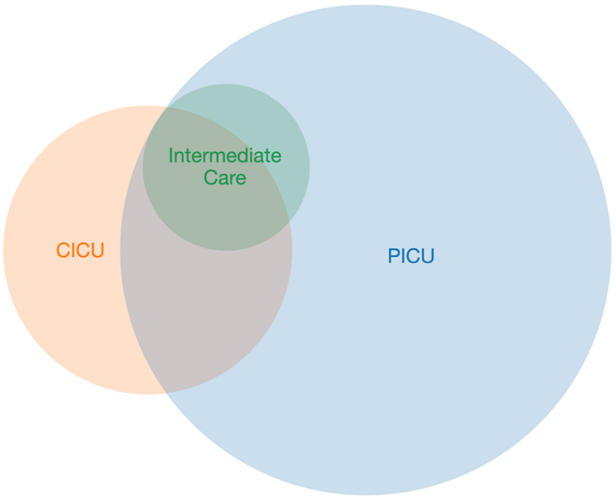

Supplementary Figure 1: Venn Diagram of the number of pediatric intensive care units (PICUs), cardiac intensive care units (CICUs) and intermediate care units, showing an overlap between 26 PICUs, 9 CICUs, and 3 intermediate care units.

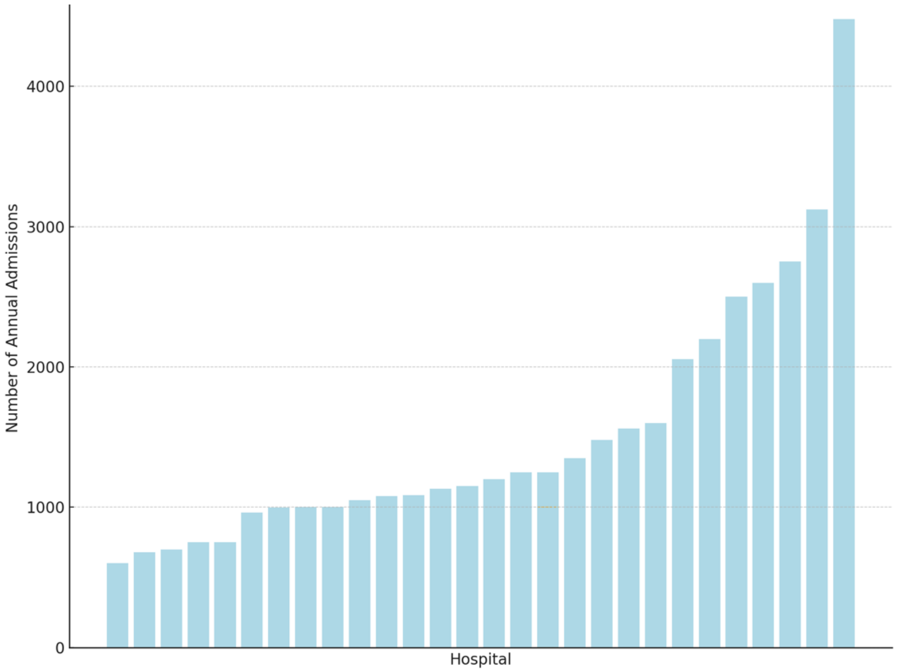

Supplementary Figure 2: Annual admissions

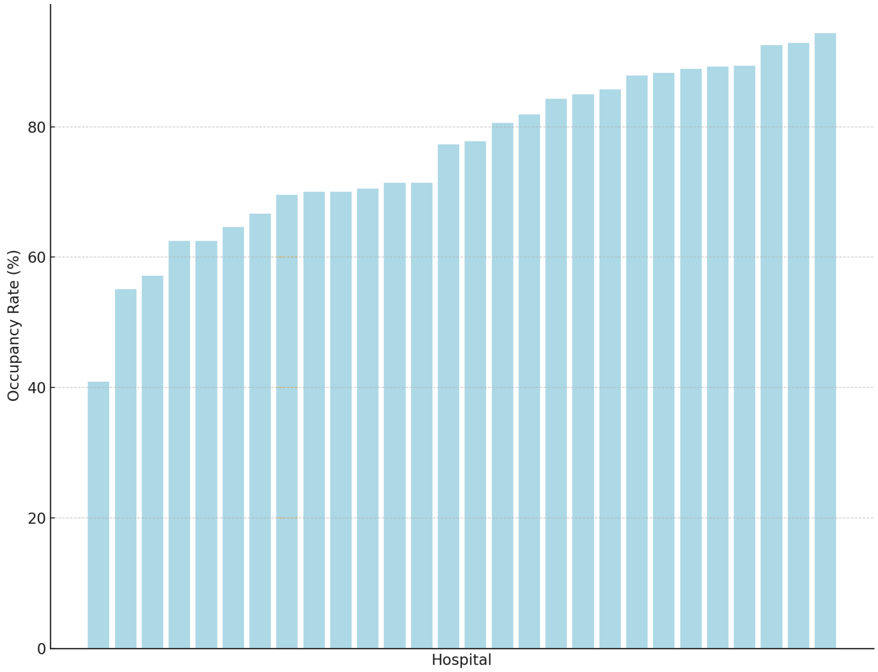

Supplementary Figure 3: Occupancy rate

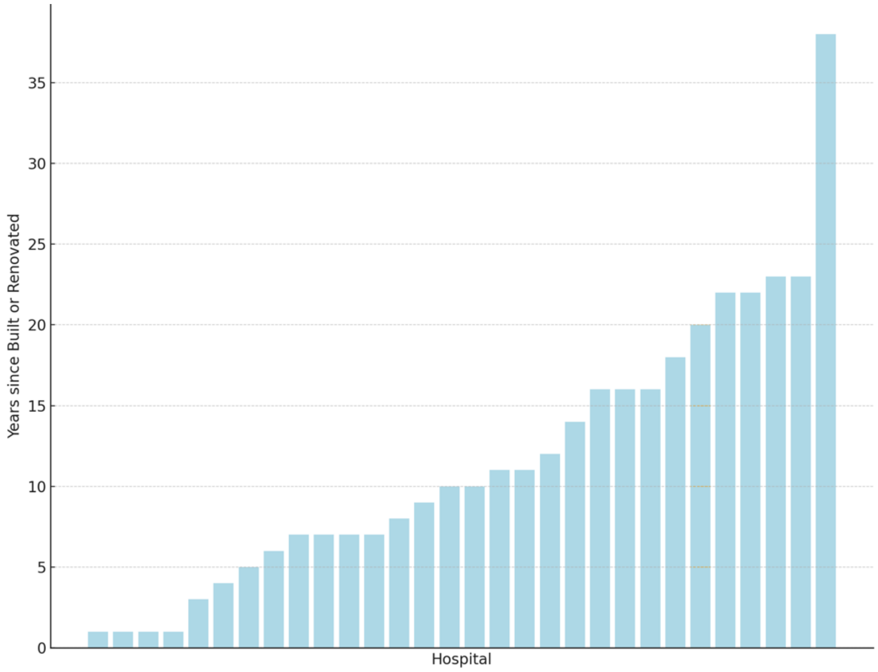

Supplementary Figure 4: Years since building or renovation

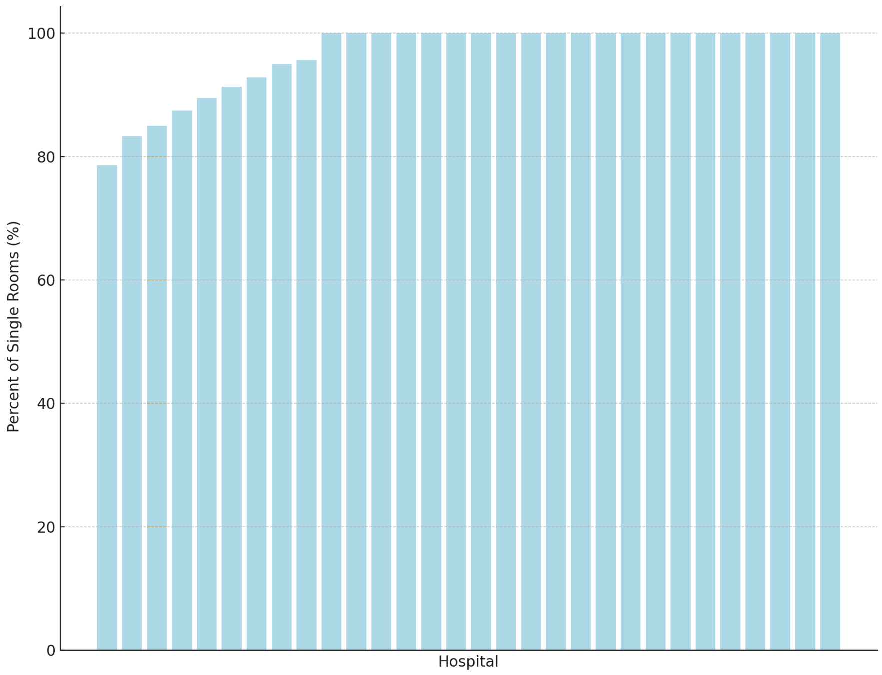

Supplementary Figure 5: Proportion of single rooms

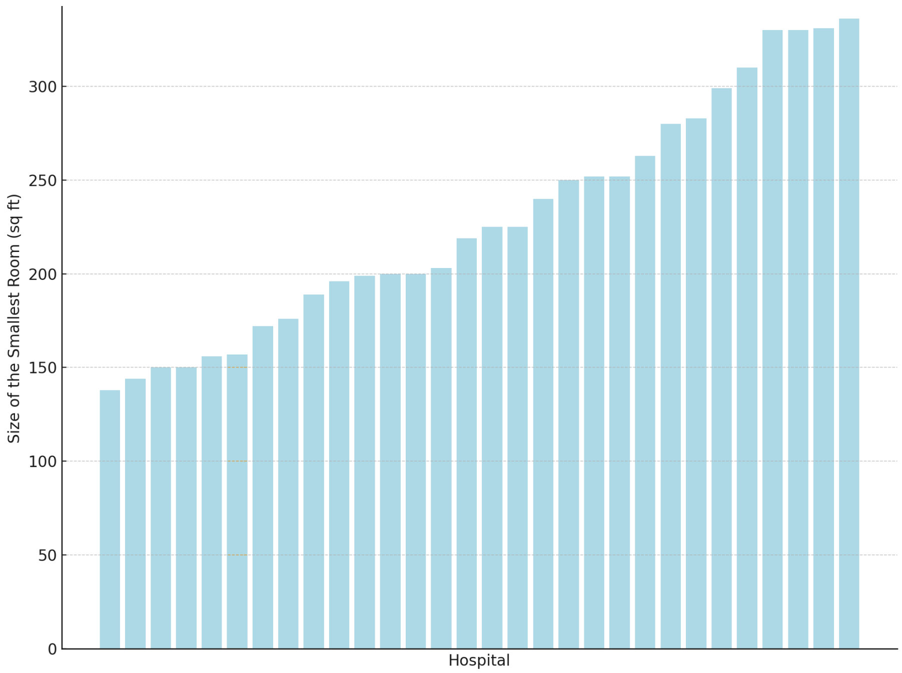

Supplementary Figure 6: Size of the smallest room

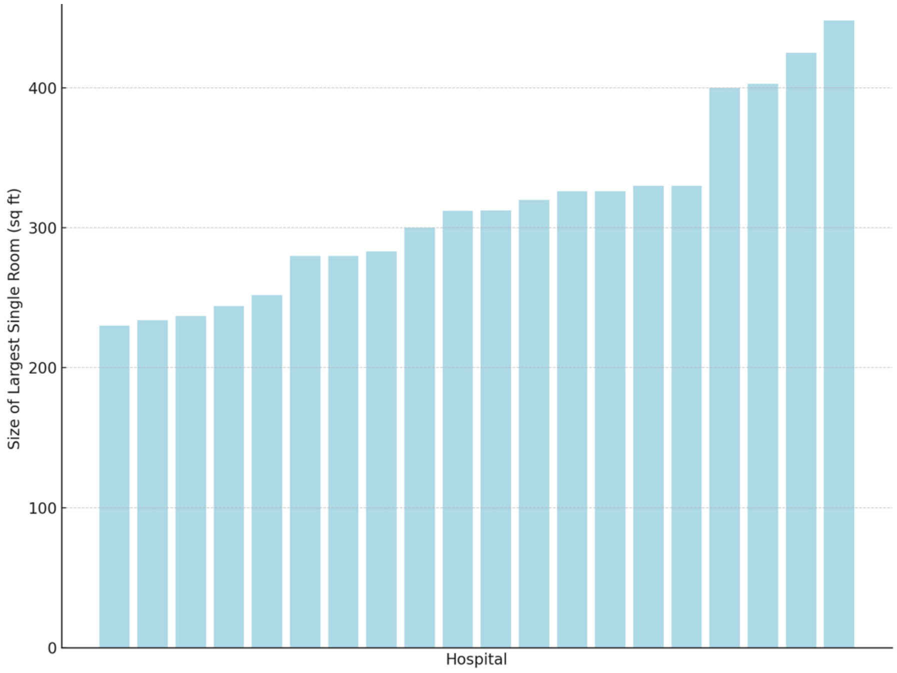

Supplementary Figure 7: Size of the largest single room

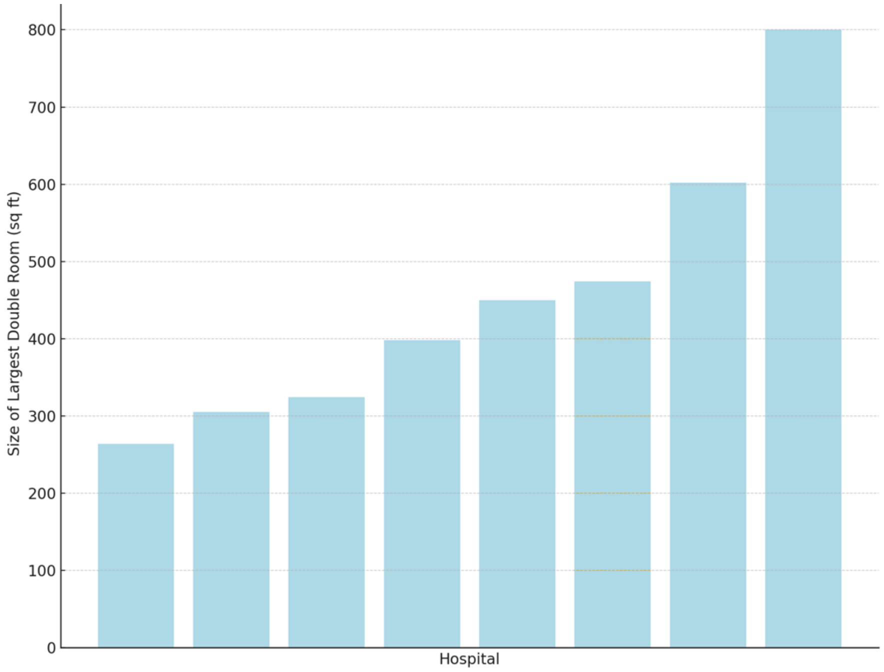

Supplementary Figure 8: Size of the largest double room

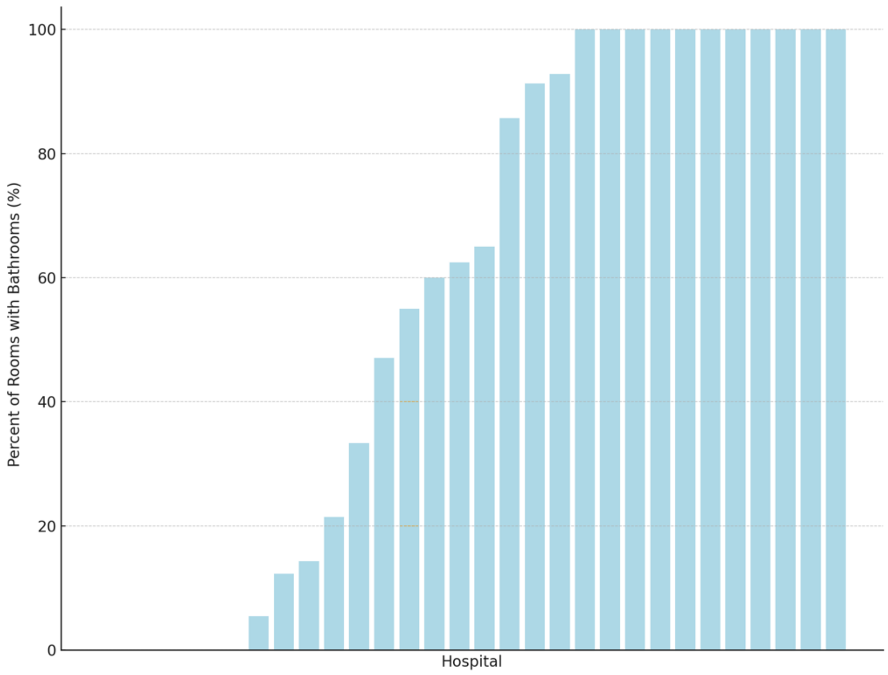

Supplementary Figure 9: Proportion of rooms with bathrooms

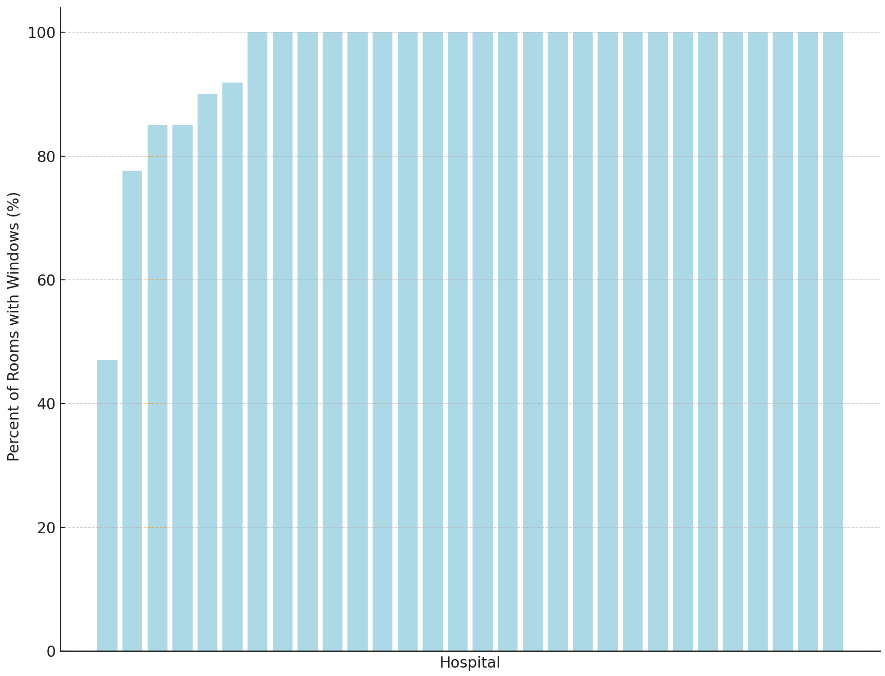

Supplementary Figure 10: Proportion of rooms with windows

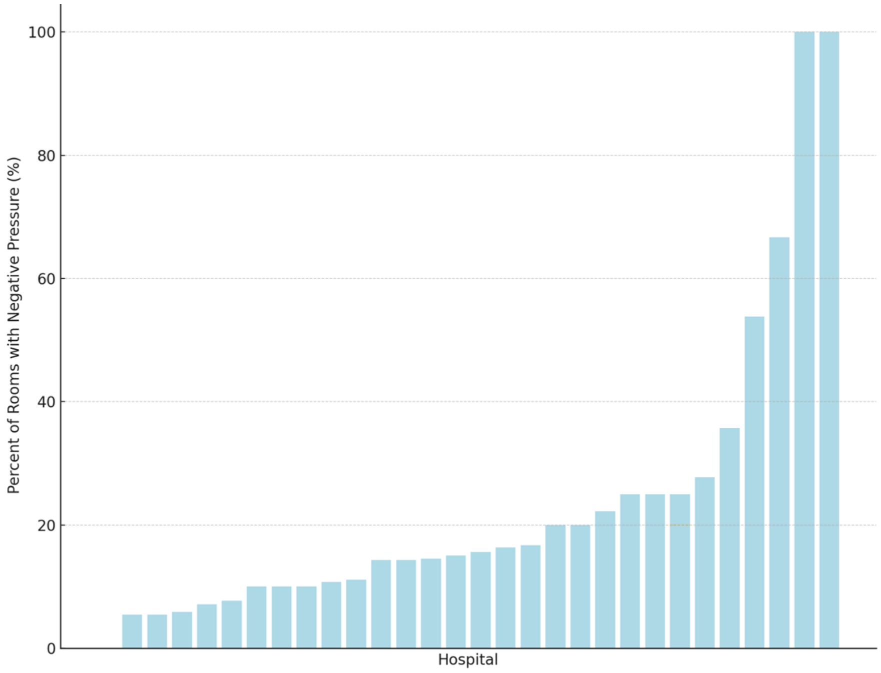

Supplementary Figure 11: Proportion of rooms with negative pressure capabilities

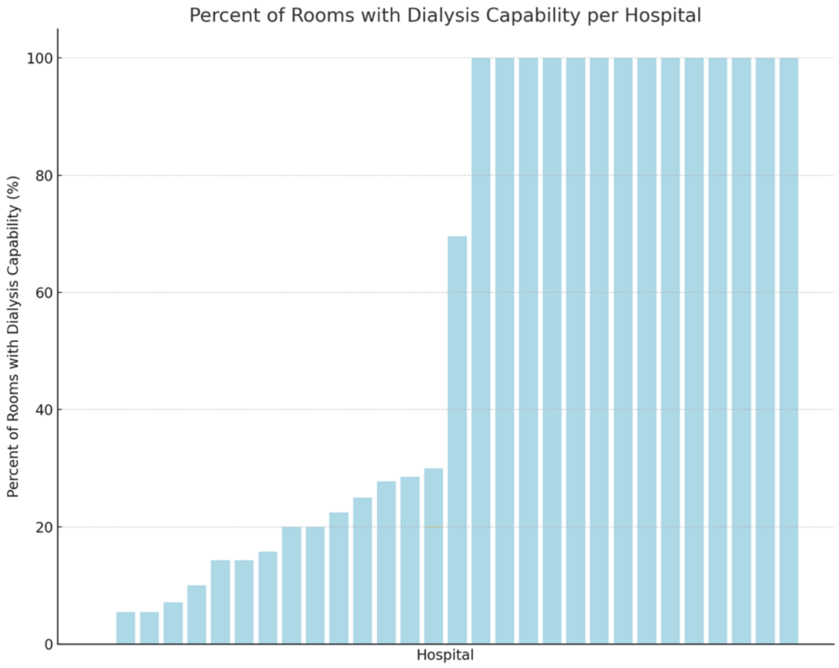

Supplementary Figure 12: Proportion of rooms with dialysis capabilities
